# Cell morphology best predicts tumorigenicity and metastasis in vivo across multiple TNBC cell lines of different metastatic potential

**DOI:** 10.1186/s13058-024-01796-8

**Published:** 2024-03-11

**Authors:** Sydney J. Conner, Justinne R. Guarin, Thanh T. Le, Jackson P. Fatherree, Charlotte Kelley, Samantha L. Payne, Savannah R. Parker, Hanan Bloomer, Crystal Zhang, Kenneth Salhany, Rachel A. McGinn, Emily Henrich, Anna Yui, Deepti Srinivasan, Hannah Borges, Madeleine J. Oudin

**Affiliations:** 1 Department of Biomedical Engineering, Tufts University, 200 College Ave, Medford, MA 02155 USA; 2 Department of Biomedical Sciences, University of Guelph, 50 Stone Rd E, Guelph, ON Canada

**Keywords:** Metastasis, Invasion, Migration, Cell lines, Adhesion, Cell lines, Triple-negative breast cancer

## Abstract

**Background:**

Metastasis is the leading cause of death in breast cancer patients. For metastasis to occur, tumor cells must invade locally, intravasate, and colonize distant tissues and organs, all steps that require tumor cell migration. The majority of studies on invasion and metastasis rely on human breast cancer cell lines. While it is known that these cells have different properties and abilities for growth and metastasis, the in vitro morphological, proliferative, migratory, and invasive behavior of these cell lines and their correlation to in vivo behavior is poorly understood. Thus, we sought to classify each cell line as poorly or highly metastatic by characterizing tumor growth and metastasis in a murine model of six commonly used human triple-negative breast cancer xenografts, as well as determine which in vitro assays commonly used to study cell motility best predict in vivo metastasis.

**Methods:**

We evaluated the liver and lung metastasis of human TNBC cell lines MDA-MB-231, MDA-MB-468, BT549, Hs578T, BT20, and SUM159 in immunocompromised mice. We characterized each cell line's cell morphology, proliferation, and motility in 2D and 3D to determine the variation in these parameters between cell lines.

**Results:**

We identified MDA-MB-231, MDA-MB-468, and BT549 cells as highly tumorigenic and metastatic, Hs578T as poorly tumorigenic and metastatic, BT20 as intermediate tumorigenic with poor metastasis to the lungs but highly metastatic to the livers, and SUM159 as intermediate tumorigenic but poorly metastatic to the lungs and livers. We showed that metrics that characterize cell morphology are the most predictive of tumor growth and metastatic potential to the lungs and liver. Further, we found that no single in vitro motility assay in 2D or 3D significantly correlated with metastasis in vivo*.*

**Conclusions:**

Our results provide an important resource for the TNBC research community, identifying the metastatic potential of 6 commonly used cell lines. Our findings also support the use of cell morphological analysis to investigate the metastatic potential and emphasize the need for multiple in vitro motility metrics using multiple cell lines to represent the heterogeneity of metastasis in vivo.

**Supplementary Information:**

The online version contains supplementary material available at 10.1186/s13058-024-01796-8.

## Background

Metastasis, the dissemination of primary tumor cells to secondary organs in the body, is the leading cause of death in breast cancer patients. In the United States, it is estimated that 287,850 women will have been diagnosed with invasive breast cancer in 2022, and 43,250 women will have died of breast cancer [1]. The 5-year survival rate for localized breast cancer is 99%, but decreases to 80% once cells have reached the lymph nodes, and drops to 29% once metastases have formed in distant organs[1]. Triple-negative breast cancer (TNBC), which represents 10–15% of all breast cancers, is known to have higher rates of metastasis and disease recurrence with distant metastases [2], with even lower 5-year survival rates 8–16% lower than non-TNBC [3]. TNBC lacks the expression of targetable receptors making it more difficult to treat [4, 5], with chemotherapy still being the standard of care for this subtype. Metastasis requires the local invasion of tumor cells into the surrounding environment, followed by entry into the lymphatics or vasculature and subsequent colonization of distant organs to form metastases [6, 7]. Metastasis occurs early on in cancer progression and significantly reduces survival in any cancer [8]. All steps of the metastatic cascade require tumor cell migration: tumor growth, local dissemination and intravasation, and colonization [9–11]. While mutations and epigenetic changes play a critical role in driving cancer progression, no single mutation renders cells metastatic or distinguishes a local from a metastatic tumor. Instead, metastasis is driven by multiple signaling pathways inside the cell in response to many different cues from the local tumor environment [12, 13]. Recent studies have suggested that chemotherapy drugs commonly used to inhibit tumor growth do not target invading cells and may promote local invasion of the surviving cells in breast tumors [14–16]. Eradicating metastatic breast cancer requires an in-depth understanding of the mechanisms that drive tumor cell migration.

In vitro*,* cell migration assays are commonly used to dissect the role of specific pathways in driving metastasis and identify drugs that could target metastatic disease by reducing cell migration. However, evaluating which cell migration assay is most informative of metastatic potential is an ongoing challenge. Many studies on cell motility have relied primarily on 2D assays where cells are seeded on a flat surface. Collective cell behaviors have been studied with the scratch wound assay, where cell migration is quantified by measuring wound closure over time, while single-cell motility behaviors can be assessed by 2D live imaging where both cell speed and persistence can be quantified [17–19]. Changes in cell morphology, driven by the cytoskeleton, are critical for cell migration. Cell morphology has been shown to be driven by distinct gene expression patterns and is a metric that can predict metastatic potential [20, 21]. We have also shown that cell morphology predicts the invasive behavior of TNBC cells in the context of extracellular matrix-driven migration [22]. 3D assays, where the cells are encapsulated in a 3D ECM scaffold, are known to better mimic the complex biochemical and physical properties of the native tissue microenvironment. Migration behavior in 2D versus 3D differs due to the more complex interactions between the microenvironment and the cytoskeleton in 3D [18, 23]. Spheroid assays, where cancer cells form aggregates encapsulated in a scaffold, have also been used to better mimic in vivo tumor dynamics [24, 25]. More complex assays involving microfluidic devices have also been developed and used to predict metastasis. Yankaskas et. al showed that the ability of cells to migrate through confined spaces, in combination with proliferation, could predict metastatic abilities in breast cancer [26]. Typically, a combination of these metrics is required by researchers to correlate or even predict in vitro cell behavior to in vivo response.

Most studies investigating factors regulating migration and metastasis rely on human breast cancer cell lines. Since the first breast cancer cell line BT20 was established in 1958 from the breast tumor of a 74-year-old female patient, numerous cell lines have been developed and used in vitro and in vivo [27, 28]. The most widely used TNBC cell line, MDA-MB-231 (231), has been cited 19,331 times on PubMed. Other commonly used cell lines are MDA-MB-468 (468), BT549, Hs578T, and SUM159 (Additional file 1: Fig. S1). While it is known that these cell lines have different properties and abilities for growth and metastasis, these cell lines' in vitro morphological, proliferative, migratory, and invasive behavior, and their correlation to in vivo behavior are poorly understood. Thus, we sought to classify each cell line as poorly or highly metastatic by characterizing tumor growth and metastasis to the lungs and livers in a murine model of six commonly used human TNBC xenografts 231, 468, BT549, Hs578T, BT20, and SUM159. The most common sites of metastasis in TNBC patients are in order: bone, lung, liver and brain [29, 30]. While bone is the most common site, even the most metastatic cell lines, like MDA-MB-231, do not metastasize to murine bone from the orthotopic site in BALB/c nude and NOD SCID mice [31], and instead require intravenous injection or subcutaneous implantation of human bones to get bone metastases [32, 33]. We then characterized each cell line's morphology, proliferation, and motility in 2D and 3D to determine the variations in these parameters between cell lines. We identify 231, 468, and BT549 cells as highly tumorigenic and metastatic, BT20 as intermediate tumorigenic, poorly metastatic to the lungs but highly metastatic to the livers, SUM159 as intermediate tumorigenic but poorly metastatic to the lungs and livers, and Hs578T as poorly tumorigenic and metastatic. Further, we show that metrics characterizing cell morphology are the most predictive of tumor growth and metastatic potential, but that no single in vitro motility assay significantly correlates with metastasis in vivo*.*

## Methods

### Animal studies

The Tufts University Institutional Animal Care and Use Committee reviewed and approved all animal studies. Female NOD/SCID mice were obtained from The Jackson Laboratory (Bar Harbor, ME). At 6–8 weeks of age, female mice were injected with 2 million cells suspended in 20% collagen I (Corning, Rat Tail Collagen I) in PBS into the fourth left mammary fat pad using a 25G needle. Mice were monitored for tumor growth for 9 weeks. Tumor burden was monitored using digital calipers each week. After 9 weeks or until the maximum tumor burden of 1.5 cm^3^ was reached or significant ulceration, mice were euthanized by CO_2_ asphyxiation and cervical dislocation. Mammary tumors or fat pads, lungs, and livers were excised for further study.

### Antibodies

Primary antibodies used in this study include: α-GAPDH (1:2500, 14C10, Cell Signaling Technology, Danvers, MA), α-Progesterone Receptor A/B (1:1000, 8757, Cell Signaling Technology), α-HER2 (1:1000, 2165, Cell Signaling Technology), α-Estrogen Receptor α (1:1000, 8644, Cell Signaling Technology), α-Vimentin (1:1000, MA5-11,883, ThermoFischer Scientific), α-Ecadherin (1:1000, ECCD-2, #13–1900, ThermoFischer Scientific) and α-Ki67 (1:1000, #9449 Cell Signaling Technology).

### Western blotting

Protein lysates were separated using SDS–polyacrylamide gel electrophoresis on a 12% polyacrylamide gel. Proteins were transferred to a nitrocellulose membrane using a TransBlot Turbo Transfer system (Bio-Rad; Hercules, CA). Membranes were blocked in 5% nonfat dry milk in tris-buffered saline with 0.05% tween 20 and incubated in primary antibody overnight at 4 ºC with rocking. Antibody binding was visualized using horseradish peroxidase-conjugated secondary antibodies (Jackson ImmunoResearch, West Grove, PA). Imaging was performed using a ChemiDoc MP imaging system (12,003,154; Bio-Rad, Hercules, CA).

### Histology

Mammary tumors, livers, and lung tissues were dissected, washed in PBS, and fixed for 24 h in 4% paraformaldehyde in PBS before tissue processing in formalin. Tissues were then embedded in paraffin and sectioned into 10 μm sections. For hematoxylin and eosin staining, sections were deparaffinized, hydrated, and stained using standard procedures with hematoxylin (GHS280; Sigma-Aldrich, St. Louis, MO), and counterstained with eosin (HT110180; Sigma-Aldrich, St. Louis, MO). Stained sections were mounted with toluene ﻿(SP15-500, Fisher Scientific, Hampton, NH). All slides were imaged using a Keyence BZ-X710 microscope (Keyence, Elmwood Park, NJ). Histologic analysis of hematoxylin and eosin (H&E) staining of these lungs and livers was quantified for metastases. Metastatic lesions were calculated by counting the number of metastases and the size of the metastasis using ImageJ.

For immunofluorescence, tissue sections were deparaffinized and antigen retrieval was performed in Citra Plus solution (HK057; Biogenex, Fremont, CA). Sections were then blocked in PBS with 0.5% tween 20 and 10% donkey serum and incubated with primary antibodies overnight at 4 °C. The next day, sections were incubated with fluorophore-conjugated secondary antibodies and 4′,6-Diamidino-2-phenylindole (DAPI; D1306; Thermo Fisher Scientific, Waltham, MA) to stain cell nuclei. Sections were mounted in Fluoromount mounting medium (00–4958-02; Thermo Fisher Scientific, Waltham, MA) and imaged using a Keyence BZ-X710 microscope (Keyence, Elmwood Park, NJ) capturing five fields of view per section. Quantification of Ki67 signal was performed by measuring area of Ki67 positivity using ImageJ (National Institutes of Health, Bethesda, MD).

### Cell culture

MDA-MB-231, MDA-MB-468, BT20, BT549, Hs578T, and SUM159, cells were obtained from American Type Cell Collection (Manassas, VA). MDA-MB-231, MDA-MB-468, and BT20 were cultured in Dulbecco’s modified Eagle’s medium (DMEM) with 10% fetal bovine serum and 1% penicillin–streptomycin-glutamine. BT-549 and Hs578T were cultured in Dulbecco’s modified Eagle’s medium (DMEM) with 10% fetal bovine serum, 1% penicillin–streptomycin-glutamine, and 10 μg/mL insulin. SUM159 were cultured in F12, 5% fetal bovine serum, 1% anti-anti, 5 μg/mL insulin, 1 μg/mL hydrocortisone, and 20 ng/mL epidermal growth factor (EGF). Fluorescently labeled cells were generated by transduction of TurboGFP-containing lentiviral particles (SHC003V; Sigma-Aldrich). Cells were monitored for mycoplasma contamination every 2–3 months by polymerase chain reaction (PCR) using the ﻿Universal Mycoplasma Detection Kit (30-1012 K; ATCC, Manassas, VA). All cells used in this study were mycoplasma negative.

### 2D cell adhesion assay

Clear plastic plates coated with 1 mg/ml Collagen I protein were seeded with either MDA-MB-231, MDA-MB-468, BT20, BT549, Hs578T, and SUM159 cells (5,000 cells/well) and left to attach for 4 or 24 h. Then cells were fixed using 4% PFA for 10 min, permeabilized with 0.2% Triton-X-100, blocked with 3% bovine serum albumin (BSA), and stained to visualize their cytoskeleton (F-actin) with phalloidin and their nuclei with DAPI. Cells were imaged using a Keyence BZ-X710 microscope (Keyence, Elmwood Park, NJ) and the morphology of each cell was quantified using Cell Profiler. Cells were first identified from the nucleus, and the outline of each cell was determined from the cytoplasm staining. Cells at the edge of an image were discarded. 2D adhesion was quantified by parameters: area/cell (number of square μm in the cell cytoplasm), compactness (mean squared distance of the cell cytoplasm from the centroid divided by the area, where a filled circle has a value of 1, and an irregular shape has a value greater than 1), eccentricity (ratio of the distance between the foci of the ellipse and its major axis length, where a perfect circle has a value of 0, and more elongated cells have a value of 1), form factor (calculated as 4πArea/Perimeter^2^, where a perfect circle has a value of 1), perimeter, and solidity (proportion of pixels that are in the convex hull that are also in the cell cytoplasm, where a perfect circle has a value of 0). Data are the result of three independent experiments with three technical replicates per experiment.

### 2D cell proliferation

Cells were seeded on Collagen I (1 mg/ml) coated 96 well polystyrene plates (5,000 cells per well) and allowed to adhere for 24 or 72 h. PrestoBlue™ Cell Viability Reagent (A13261; Invitrogen, Carlsbad, CA) was added to each well according to the manufacturer’s instructions and incubated for 30 min at 37 °C. The absorbance of cells plated for 24 h and 72 h was measured on a plate reader and the fold change in absorbance was quantified between 24 and 72 h.

### 2D cell migration

MDA-MB-231, MDA-MB-468, BT20, BT549, Hs578T, and SUM159 cells transfected with GFP were plated (5,000 cells/well) on plates coated with 1 mg/ml Collagen I protein for 1 h and allowed to adhere for 2 h at 37 °C before imaging. Cells were imaged every 20 min overnight for 16 h using a Keyence BZ-X710 microscope (Keyence, Elmwood Park, NJ). Cells were then tracked using VW-9000 Video Editing/Analysis Software (Keyence, Elmwood Park, NJ), and migration speed and persistence were calculated using a custom MATLAB script vR2019a (MathWorks, Natick, MA).

### 3D single cell invasion

MDA-MB-231, MDA-MB-468, BT20, BT549, Hs578T, or SUM159 cells transfected with GFP were resuspended in full media (400,000 cells/ml) with 1 mg/mL Collagen I (354,236; Corning, Corning, NY), 10 mM NaOH, 7.5% 10X DMEM, and 50% DMEM and plated in 96 well glass bottom plates. Plates were incubated at 37 °C for 2 h before imaging. Once ECM gelled, 50 μL of culture media was added. Z-stack images of cells were captured every 20 min for 16 h using the Keyence BZ-X710 microscope (Keyence, Elmwood Park, NJ). Cells were then tracked using VW-9000 Video Editing/Analysis Software (Keyence, Elmwood Park, NJ), and invasive speed and persistence were calculated using a custom MATLAB script vR2019a (MathWorks, Natick, MA).

### 3D spheroid invasion

MDA-MB-231, MDA-MB-468, BT20, BT549, Hs578t, and SUM159 cells transfected with GFP were seeded with full media in round-bottom, low-attachment 96-well plates (1,000 cells/well) and centrifuged at 3,000 rpm for 3 min to form spheroids. Spheroids were incubated at 37 °C for three days. After 3 days spheroids were either grown in media or an ECM mixture of 1 mg/mL Collagen I (354,236; Corning, Corning, NY), 10 mM NaOH, 7.5% 10X DMEM, and 50% DMEM. Once ECM gelled, 50uL of culture media was added. Spheroids were imaged using a Z-stack on the day of ECM addition (day 0) and after 4 days of growth using a Keyence BZ-X710 microscope (Keyence, Elmwood Park, NJ). Spheroid invasion was quantified by measuring the distance cells on the periphery of the spheroid invade from the center and averaging for each spheroid. The fold change in spheroid invasion distance was then quantified relative to the average invasion distance of no ECM control spheroids for each cell line. The data presented are the result of three independent experiments with three technical replicates per experiment.

### Statistical analysis

GraphPad Prism v9.1.0 was used for the generation of graphs and statistical analysis. Significance was determined using a one-way ANOVA with Turkey’s multiple comparisons test unless otherwise stated. MATLAB was used to determine linear relationships and correlation.

## Results

### Characterization of tumor growth and metastasis of cell line xenografts in vivo

**Fig. 1:**
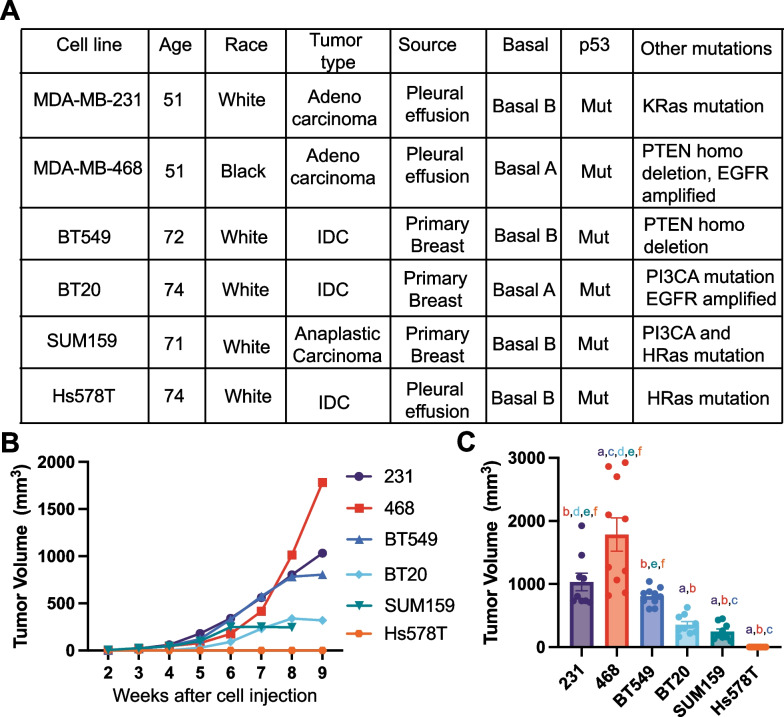
Tumor growth of human TNBC cell xenografts implanted in immunocompromised female NOD-SCID mice*.* **A** Characteristics of six human TNBC cell lines used in the study. IDC, invasive ductal carcinoma; **B** Tumor volume over time for each cell line 231, 468, BT549, Hs578T, BT20, and SUM159. **C** Tumor volume at the terminal endpoint. Data are shown as mean with SEM, n = 8–10 animals/group. Significance was determined using a one-way ANOVA with Tukey’s multiple comparison test comparing each cell line to every other cell line. Significance (*p* < 0.05) is denoted by a letter corresponding to each cell line tested, 231 (a), 468 (b), BT549 (c), BT20 (d), SUM159 (e), Hs578T (f)

There are currently no studies that directly compare the metastatic ability of multiple TNBC cell lines with the same murine xenograft model and experimental parameters. We first directly compared the growth and metastasis of six human TNBC cell lines commonly used to study mechanisms of TNBC metastasis. We used 231, 468, BT549, Hs578T, and SUM159 cell lines, which were isolated from patients with different racial backgrounds and tumor types and which have different mutational profiles and properties (Fig. 1A). We confirmed the receptor status of all 6 cell lines, showing that all cell lines have low levels of HER2, and no expression of progesterone receptor or estrogen receptor α (Additional file 1: Fig S1B). All cell lines except 468 expressed Vimentin and no E-cadherin (Additional file 1: Fig S1C), suggesting most cell lines are more mesenchymal, while 468 are more epithelial, in line with previous reports [34]. We injected the same concentration of cells in a Collagen I solution into the 4th left mammary fat pad of female NOD-SCID mice. Tumors were left to grow for 9 weeks or until ulcers began to form. We found that 468 tumors grew the fastest, followed by 231 and BT549 tumors which had similar sizes. BT20 and SUM159 xenografts grew the smallest tumors, and at 8 weeks the SUM159 tumors had ulcerated and had to be sacrificed (Fig. 1B, C, Additional file 1: Fig S2). The Hs578T xenografts did not grow palpable tumors in this time frame. Based on the tumor latency (Additional file 1: Fig S2G) and the endpoint tumor size (Fig. 1C), the data suggest that 231, 468, and BT549 cell lines are highly tumorigenic, BT20 and SUM159 are intermediate tumorigenic, and Hs578T are poorly tumorigenic since they did not form palpable tumors.

**Fig. 2:**
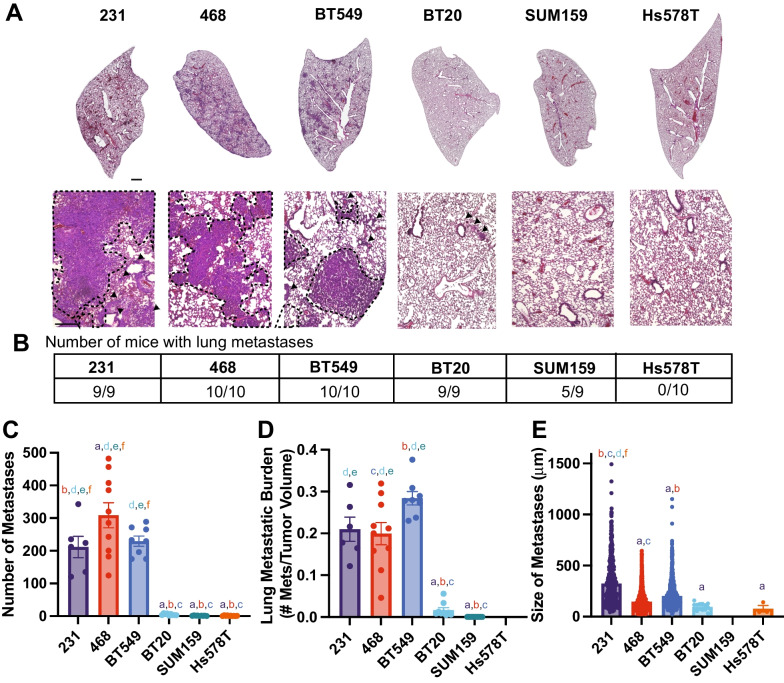
TNBC cells have different metastatic potentials to the lung **A** Representative tissue sections stained with H&E, obtained from lungs of mice 9 weeks after injection of TNBC cells in their 4th left mammary gland. Scale bar = 500 μm Inset scale bar = 500 μm. **B** Number of mice that developed lung metastases for each cell line. **C** The number of metastases per lung for each cell line and **D** Metastatic burden in lungs (ratio of the number of metastases in the lungs to the primary tumor volume) **E** Average size of each lung metastasis. Data are shown as mean with SEM 231 n = 6, 468 n = 10, BT549 n = 8, BT20 n = 9, SUM159 n = 9, Hs578T n = 10 animals/group. Significance was determined using a one-way ANOVA with Tukey’s multiple comparison test comparing each cell line to every other cell line. Significance (*p* < 0.05) is denoted by a letter corresponding to each cell line tested, 231 (a), 468 (b), BT549 (c), BT20 (d), SUM159 (e), Hs578T (f)

Next, we examined lung and liver metastases, by quantifying the number of metastases found in each organ, the average size of each metastatic lesion, and the metastatic index, which is the number of metastases relative to primary tumor size. The 231, 468, and BT549 cell lines had the most lung metastases and similar metastatic index. The BT20, Hs578T, and SUM159 cells had very low numbers of lung metastases, with very low metastatic index (Fig. 2A-D). The size of lung metastases varied between cell lines with 231 s having significantly larger metastases than BT549 and 468 (Fig. 2E). The lung metastatic lesions in BT20, Hs578T, and SUM159 were very small (Fig. 2E). We then examined the relationship between primary tumor size, number of lung metastases, and size of lung metastases. For all cell lines, there was a significant correlation between primary tumor volume and the number of metastatic lesions in the lung (Additional file 1: Fig S3A, B). There was no significant correlation between the number and sizes of lung metastases (Additional file 1: Fig S3C, D) or between the size of the primary tumor and the size of the metastases in the lungs (Additional file 1: Fig S3E, F).

**Fig. 3:**
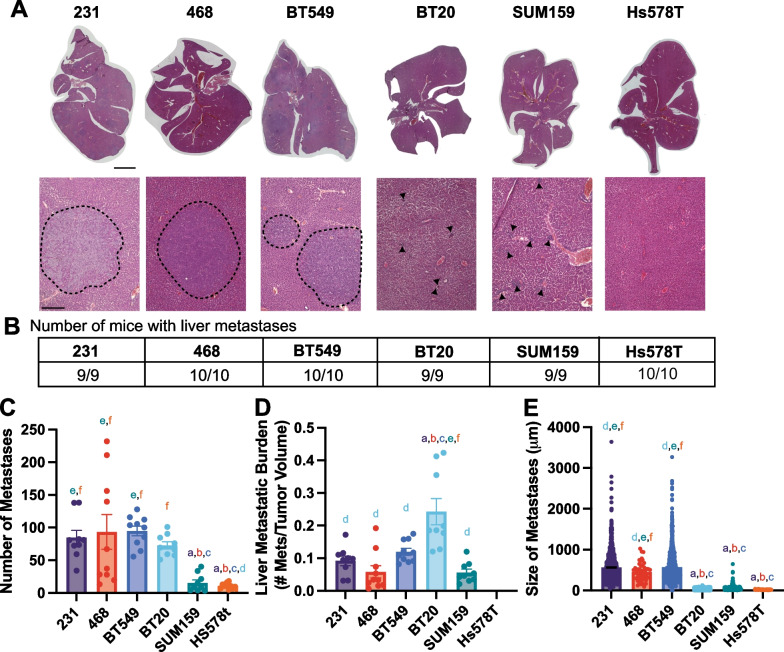
TNBC cells have different metastatic potentials to the liver **A** Representative tissue sections stained with H&E, obtained from livers of mice 9 weeks after injection of TNBC cells in their 4th left mammary gland. Scale bar is 1 mm; inset scale bar is 500 μm dashed lines represent metastasis boundary, black arrows identify smaller metastases. **B** Number of mice that developed liver metastases for each cell line. **C** The number of metastases per liver for each cell line and **D** Metastatic burden in the liver (ratio of the number of metastases in the lungs to the primary tumor volume) **E** Average size of each liver metastasis. Data are shown as mean with SEM 231 n = 9, 468 n = 10, BT549 n = 10, BT20 n = 9, SUM159 n = 10, Hs578T n = 10 animals/group. Significance was determined using a one-way ANOVA with Tukey’s multiple comparison test comparing each cell line to every other cell line. Significance (*p* < 0.05) is denoted by a letter corresponding to each cell line tested, 231 (a), 468 (b), BT549 (c), BT20 (d), SUM159 (e), Hs578T (f)

The BT549, 231, and 468 cell lines developed the most liver metastases, with similar levels of metastatic index and size of metastases (Fig. 3A–D). The BT20 xenografts resulted in a high number of liver metastases, which given their small tumors, led to a high liver metastatic index for this cell line, significant relative to 231 cells (Fig. 3A–E). Interestingly, for the BT20 cell line while the number of liver metastases was not significantly different from the 231 cell line the size of the metastases was significantly smaller (Fig. 3A–E). SUM159 and Hs578T xenografts both developed significantly fewer liver metastases than 231s (Fig. 3A–E), which were also smaller. We then examined the relationship between primary tumor size, number of liver metastases, and size of liver metastases. In the livers, there were differences between the highly metastatic and poorly metastatic cell lines. There was a significant correlation between primary tumor volume and the number of metastatic lesions in the liver, and the number and size of liver metastases but only for the least metastatic cell lines (BT20, Hs578T, and SUM159) (Additional file 1: Fig S3G-J). For the highly metastatic cell lines (231, 468, and BT549), there was a negative significant correlation between primary tumor size and liver metastases size (Additional file 1: Fig S3E, F).

These data characterize the tumorigenicity and metastatic potential of six human TNBC cell lines, demonstrating that 231, 468, and BT549 cell lines are highly tumorigenic, BT20 and SUM159 are intermediate tumorigenic, and Hs578T are poorly tumorigenic since they did not form palpable tumors. These data characterize 231, 468, and BT549 cell lines as highly metastatic to both the lungs and the livers, BT20 as poorly metastatic to the lungs but metastatic to the liver, and SUM159 and Hs578Ts as poorly metastatic to both the lungs and the liver with slight dissemination to the liver.

### TNBC cell lines have distinct differences in morphological characteristics

**Fig. 4:**
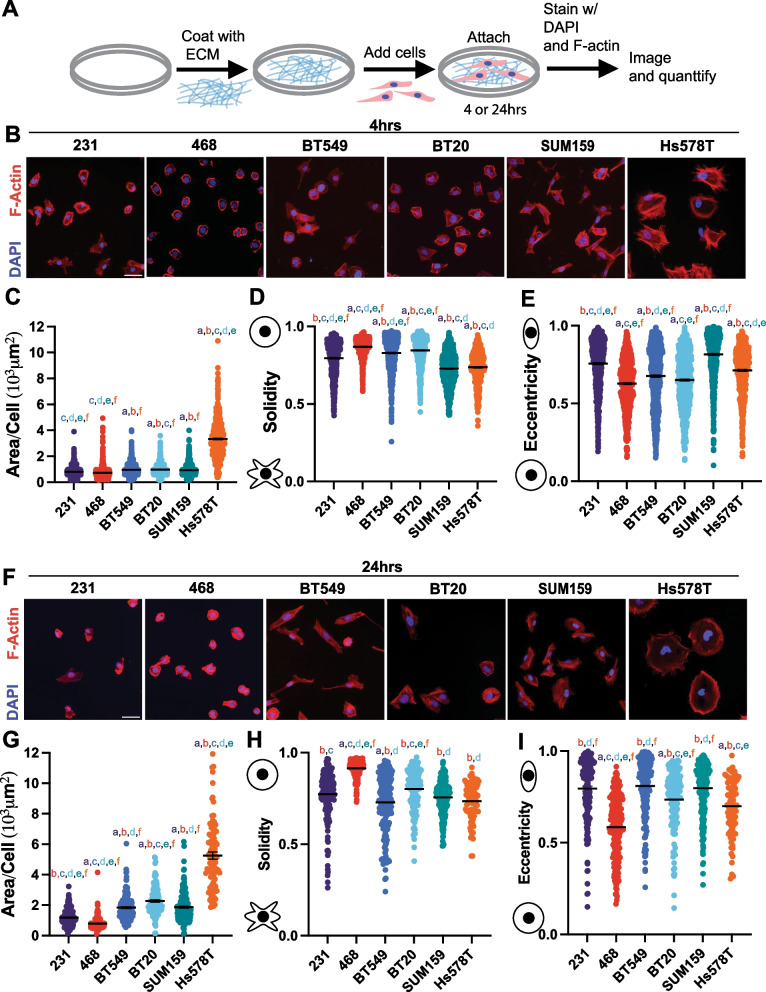
Characterization of TNBC cell line morphology. **A** Schematic depicting experimental procedure. **B** Representative images of 231, 468, BT549, BT20 SUM159 and Hs578T cell lines plated on Collagen I ECM for 4 h, fixed and stained for nuclei (Blue) and F-actin (red). Scale bar = 50 μm. Quantification of cell shape parameters for each cell line 231 (n = 679 cells), BT549 (n = 799 cells), Hs578T (n = 538 cells), 468 (n = 818 cells), BT20 (n = 882 cells) and SUM159 (n = 892 cells) **C** Area/Cell, **D** Solidity, and **E** Eccentricity. **F** Representative images of 231, BT549, Hs578T, 468, BT20, and SUM159 cell lines plated on Collagen I for 24 h, fixed and stained for nuclei (Blue) and F-actin (red). Scale bar = 50 μm. Quantification of cell shape parameters for each cell line 231 (n = 178 cells), BT549 (n = 184 cells), Hs578T (n = 91 cells), 468 (n = 205 cells), BT20 (n = 135 cells) and SUM159 (n = 192 cells) **G **Area/Cell, **H** Solidity, and **I** Eccentricity. Data show mean ± SEM. Significance was determined using a one-way ANOVA with Tukey’s multiple comparison test comparing each cell line to every other cell line. Significance (p < 0.05) is denoted by a letter corresponding to each cell line tested, 231 (a), 468 (b), BT549 (c), BT20 (d), SUM159 (e), Hs578T (f)

We next investigated the in vitro characteristics of six TNBC cells. It has been previously shown that features of cell morphology correlate to cell motility and metastatic potential [20, 21]. 2D cell adhesion assays are commonly used to investigate cell phenotypic behavior because they are relatively inexpensive and have low equipment requirements. Cells were left to adhere on either Collagen I, the most abundant ECM protein in breast tissue [35, 36], or tissue culture plastic for 4 or 24 h, and their morphology was analyzed: size (cell area and perimeter), irregularity (solidity and form factor), and elongation (eccentricity and compactness) were quantified (Fig. 4A). On Collagen I for 4 and 24 h the 468 cells were the smallest, as measured by cell area and perimeter (Fig. 4B, G, Additional file 1: Fig S4A, D), they were also least irregular indicating fewer protrusions measured by solidity and form factor, but also rounder and less elongated, measured by eccentricity and compactness. (Fig. 4D, E, H, I, Additional file 1: Fig S4B, S4C, S4E, S4F). Interestingly on uncoated plastic for 4 h 231s were smaller than 468s but after 24 h 231s and 468s were not significantly different in size (Additional file 1: Fig S5 A, S5D, S5G, S5K). After 4 h on plastic 468 s exhibited an irregular and elongated morphology, however, after 24 h they became less irregular and more round, similar to when cultured on Collagen I (Additional file 1: Fig S5 B, C, E, F, H, I, K, L). On Collagen I BT549 and BT20 cells were intermediate in size, after 4 h they both exhibited a more rounded morphology, but after 24 h BT549s were more irregular and elongated(Fig. 4B–I, Additional file 1: Fig S4) with a similar effect on plastic (Fig S5). On Collagen I for 4 and 24 h we found that Hs578Ts were the largest cells with more irregular shapes indicating more protrusions and were also rounder and less elongated (Fig. 4B-I, Additional file 1: Fig S4). However, on plastic SUM159s were the largest after 4 h but Hs578Ts were the largest after 24 h similar to on Collagen I (Additional file 1: Fig S5). SUM159 cells on Collagen I were intermediate in size but were similarly more protrusive and more elongated (Fig. 4C–I Additional file 1: Fig S4). SUM159s were very irregular, with more protrusions, and were the most elongated (Fig. 4C–I, Additional file 1: Fig S4). While the cell shape did vary between 4 and 24 h, the relative differences between the different cell lines did stay consistent.

### Proliferation and 2D cell motility

**Fig. 5:**
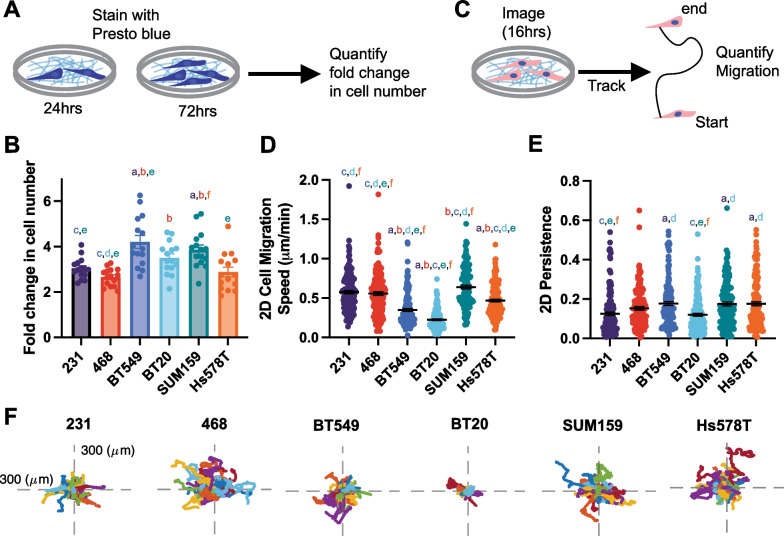
Cell line-specific proliferation, 2D migration, and persistence. **A** Schematic depiction of presto blue proliferation assay. **B** The relative proliferation of cell lines **C** Schematic depiction of 2D migration assay **D** 2D cell migration and **E** 2D persistence of cell lines seeded on Collagen 1 coated glass bottom plates. **F** representative rose plots of the migration of cells on Collagen I ECM-coated glass coverslips. Data show mean ± SEM. Significance was determined using a one-way ANOVA with Tukey’s multiple comparison test comparing each cell line to every other cell line. Significance (*p* < 0.05) is denoted by a letter corresponding to each cell line tested, 231 (a), 468 (b), BT549 (c), BT20 (d), SUM159 (e), Hs578T (f)

Cell proliferation is critical to support both primary and secondary tumor growth. We quantified the proliferation rate of each cell line by measuring the change in metabolic activity over 48 h (Fig. 5A). BT549 and SUM159 cell lines were significantly more proliferative than 231 s (Fig. 5B). 2D cell migration is a commonly used metric to determine metastatic potential. Cells were seeded on Collagen I and imaged to quantify 2D cell migration speed, which measures how fast the cell is moving over the distance traveled, and persistence which measures the Euclidean distance between the start and finish of the cell's path over the total distance traveled (Fig. 5C). SUM159, 231, and 468 cells migrated the fastest in 2D and were not significantly different from each other. Hs578T, BT20, and BT549 cells all migrated significantly slower than 231 cells (Fig. 5D). 231, 468 and BT20 cells had the lowest 2D persistence with BT594s, Hs578Ts and SUM159s all having significantly higher persistence than 231 s respectively (Fig. 5E,F).

### 3D methods to quantify cell invasion

**Fig. 6:**
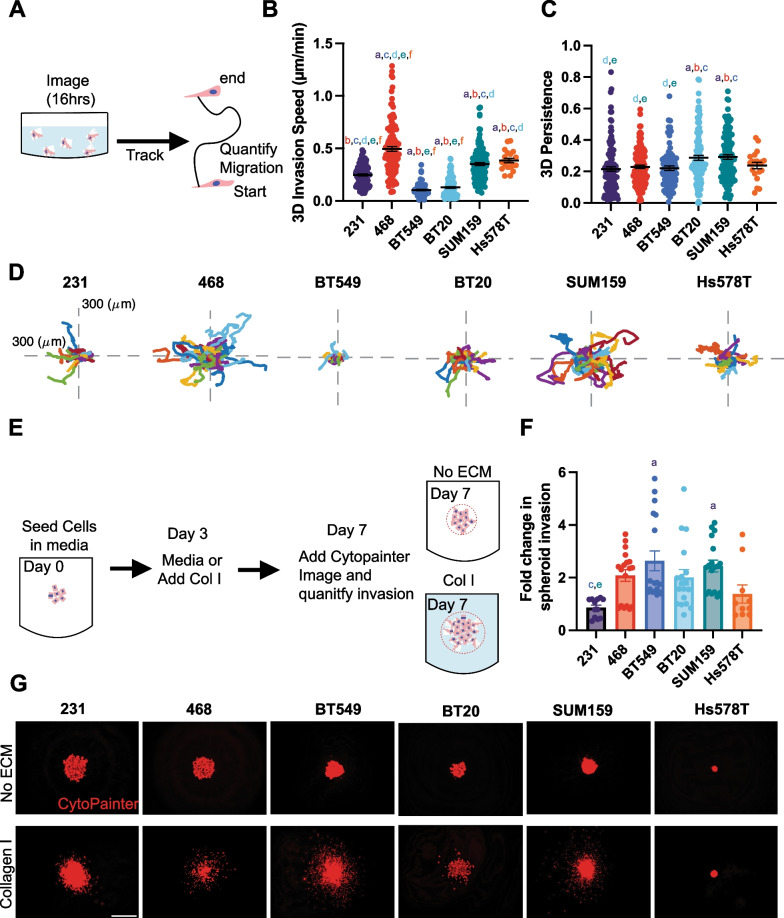
3D single cell invasion and spheroid invasion in Collagen I ECM **A** Schematic depicting experimental set up of 3D invasion assay. **B** 3D invasion of breast cancer cell lines invading through Collagen I ECM **C** 3D persistence of cells invading through collagen I ECM **D** Representative rose plots of the invasion of cells through the collagen I ECM. **E** Schematic depiction of spheroid invasion assay. **F** Fold change in spheroid invasion into Collagen I hydrogels over invasion into media **G** Representative spheroid images of 231, BT549, Hs578T, and SUM159 spheroids cultured in media (upper) or spheroids cultured in Collagen I solution visualized with cytopainter (red). Scale bar = 500 μm. Data show mean ± SEM. Significance was determined using a one-way ANOVA with Tukey’s multiple comparison test comparing each cell line to every other cell line. Significance (*p* < 0.05) is denoted by a letter corresponding to each cell line tested, 231 (a), 468 (b), BT549 (c), BT20 (d), SUM159 (e), Hs578T (f)

To better recapitulate the native breast microenvironment, 3D assays where breast cancer cells are encapsulated in ECM, are used to mimic the 3D in vivo environment. We first investigated 3D invasion in a single-cell invasion assay, where each cell line was encapsulated in Collagen I ECM and plated in a glass bottom dish then imaged overnight to track cell invasion (Fig. 6A). The 468, SUM159, and Hs578T cells invaded significantly faster than the 231 cells, and BT549 and BT20 cells invaded significantly slower than the 231 cells (Fig. 6B). Interestingly, only SUM159 and BT20 cells were significantly more persistent than 231 cells (Fig. 6 C, D).

We then used a 3D spheroid assay, where the cell lines were seeded in low attachment U-bottom dishes and centrifuged to form a densely packed spheroid. After growing for 3 days, the spheroids were either left in the original media or Collagen I was added before an additional 4 days of culture. Growth out of spheroids relies on tumor cell proliferation and invasion. We then measured the fold change in the spheroid outer area in the Collagen I group relative to media only after 7 days (Fig. 6E). 468, BT20, BT549, and SUM159 cells had a significantly higher fold change in spheroid invasion compared to 231s (Fig. 6F-G).

PCA was then used to reduce the dimensionality of the data set to compare 2D migration and persistence and 3D invasion and persistence. The scores of each cell line demonstrate that highly tumorigenic and metastatic cell lines, 231, 468, and BT549 clustered together and the poorly tumorigenic and metastatic SUM159, Hs578T, and BT20 clustered together indicating that highly and poorly tumorigenic and metastatic cell lines have similar motility characteristics (Additional file 1: Fig S6A). The loadings scores represent variations in the motility metrics. Interestingly, 2D migration and persistence and 3D invasion cluster together while 3D persistence is by itself.

### Cell shape is most correlated with metastatic potential

Our last goal was to determine which assays and their metrics, cell morphology, proliferation, and 2D or 3D motility, best correlate with in vivo tumor growth and metastasis. To do this we used pairwise comparisons to systematically investigate each in vitro metric in relation to in vivo response for all cell lines. First, we determined if there was a linear relationship between each in vitro metric and in vivo response by calculating the R^2^ which measures the proportion of variation in the in vivo response that is attributed to each in vitro metric, and the Pearson correlation which measures the strength of the linear relationship. First, comparing only cell morphology metrics on Collagen I and plastic for 4 and 24 h we found that form factor and solidity, which quantify how irregular or protrusive a cell is, correlated the strongest with metastasis to the lungs and liver and tumor volume regardless of culturing method (Additional file 1: Fig S7A-C). We also used partial least squares to calculate the scores and loadings which capture the covariance between the independent and dependent variables [37]. The scores plot describes how each cell line projects on principal components 1 (PC1) and PC2. Interestingly, we found that 231s, BT549s and BT20s clustered together (Additional file 1: Fig S7D). The loading scores represent how the independent variables and dependent in vivo responses project on each principal component(Additional file 1: Fig S7 E). We found that solidity and form factor on Collagen I for 4 h clustered closely with the in vivo response metrics. Thus, for comparing in vivo responses to all the in vitro metrics we only included the cell adhesion on Collagen I for 4 h data.

**Fig. 7:**
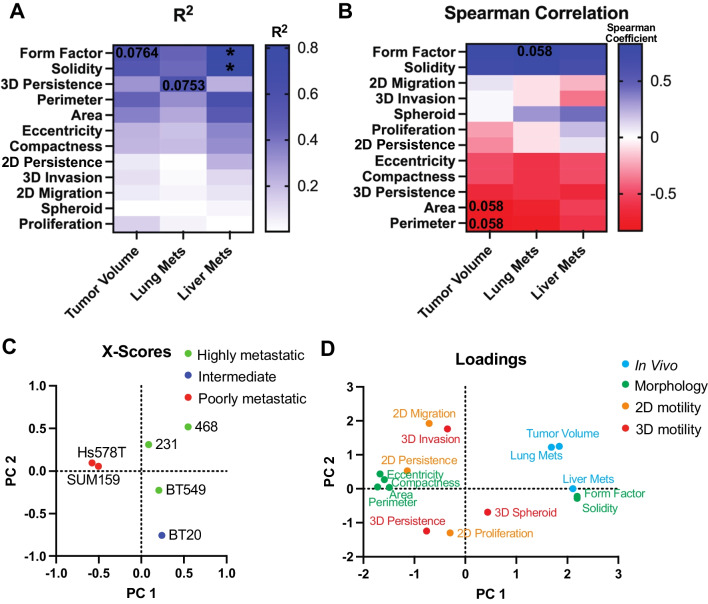
Distinct differences in cell line-specific behavior in vitro and in vivo*.* **A** pairwise analysis of the strength of the linear relationship (R^2^) between in vitro metrics and in vivo behavior. Numbers represent p-value and colors represent R^2^ value where blue is high and white is low indicated by gradient **B** Spearman correlation between in vitro metrics and in vivo behavior. Numbers represent p-value and colors represent the Spearman correlation coefficient where blue is a high positive correlation, white is no correlation and red is a negative correlation indicated by gradient **C** X-scores plot of the PLS model **D** PLS loadings for adhesion metrics, proliferation, 2D and 3D motility, and in vivo tumor volume, lung and liver metastases

We found that there were distinct differences in the linear relationship between in vitro metrics and in vivo behaviors. Interestingly, we found that solidity and form factor, which quantify how irregular or protrusive a cell is, had a linear relationship with tumor volume (R^2^ > 0.5) (Fig. 7A, Additional file 1: Fig S8). Only the persistence of 3D single cell invasion had a linear relationship to the number of lung metastases (R^2^ > 0.5) (Fig. 7A, Additional file 1: Fig S8). Cell size and cell irregularity, all had linear relationships to the number of liver metastases with solidity and form factor having a strong linear relationship (R^2^ > 0.7 and p < 0.05) (Fig. 7A, Additional file 1: S8). Next, we used Spearman's correlation to investigate the correlation of each assay to in vivo response. We found that area, perimeter, and 3D persistence were negatively correlated with tumor volume, while solidity and form factor were positively correlated (Spearman Coefficient >  ± 0.6). Area, perimeter, eccentricity, compactness, and 3D persistence demonstrated a negative correlation with the number of lung metastasis while solidity and form factor demonstrated a positive correlation. Perimeter and 3D persistence negatively correlated to the number of liver metastases, while solidity and form factor positively correlated (Fig. 7B).

To visualize the relationship between the cell lines and assays we used partial least squares to calculate the scores and loadings which capture the covariance between the independent and dependent variables [37]. The scores plot describes how each cell line projects on principal components 1 (PC1) and PC2. Highly metastatic cell lines 231, 468, and BT549 clustered together, and poorly metastatic cell lines SUM159s and Hs578T clustered together. BT20s, which were intermediate tumorigenic but poorly metastatic to the lungs but metastatic to the livers, stood alone (Fig. 7C). The loading scores represent how the independent variables and dependent in vivo responses project on each principal component. As expected, cell shape parameters related to size, elongation, and irregularity clustered together. Other cluster groups include lung metastasis and tumor volume; liver metastasis, form factor, and solidity; eccentricity, compactness, area, perimeter, and 2D persistence; 2D migration and 3D invasion; 2D, 3D persistence and proliferation (Fig. 7D). This analysis demonstrates that there were distinct differences in cell line-specific behaviors in vitro and in vivo and that parameters that measure cell shape and 3D persistence are most correlated with in vivo behaviors.

## Discussion

Established human breast cancer cell lines and murine xenograft models are powerful tools in breast cancer research that provide a comprehensive picture of disease progression and the metastatic cascade. However, there is significant heterogeneity within cell lines in terms of their behaviors both in vitro and in vivo. In vitro*,* methods are preferred for investigating mechanisms, identifying therapeutic targets, and testing hypotheses before moving to costly and time-consuming in vivo models. As such, morphology, proliferation, migration, and invasion assays are often employed to characterize a cancer cell’s potential to grow and metastasize. Yet how metrics quantified in these assays correlate with in vivo behaviors such as tumor growth and metastasis is poorly understood. In this study, we characterized the metastatic potential of 6 commonly used human TNBC cell lines both in vitro and in vivo. We generated murine xenografts to quantity tumor growth, and lung and liver metastasis and performed several in vitro assays used to study cell behaviors such as morphology, proliferation, migration, and invasion, which are all mechanisms critical for tumor growth and metastasis. We then conducted analyses to better understand how the in vitro morphological, proliferative, migratory, and invasive behavior of TNBC cell lines correlate to their in vivo behavior to determine which assays best predict tumor growth and metastasis. We identified 231, 468, and BT549 cell lines as highly tumorigenic, BT20 and SUM159 as intermediate tumorigenic, and Hs578T as poorly tumorigenic. We identified 231, 468, and BT549 cell lines as highly metastatic to both the lungs and the livers, BT20 as poorly metastatic to the lungs but highly metastatic to the liver, and SUM159 and Hs578Ts as poorly metastatic to both the lungs and the liver. We found a positive correlation between primary tumor volume and both liver and lung metastasis for most of the cell lines. This is in line with patient data, where multiple studies have shown that primary tumor volume is an independent prognostic factor for distant metastasis and poor disease survival [38, 39]. We determined that parameters measuring cell shape and 3D persistence are most correlated with in vivo behaviors.

When studying the role of a particular step in metastasis or the effect of a drug or intervention on metastasis in vivo, it is important to consider the metastatic potential of the specific cell lines being used, as well as using multiple cell lines of different metastatic potential to cover a range of human phenotypes to ensure reproducible findings. One common approach involves using TNBC cells as a highly metastatic model, ER-positive MCF7 cells as a poorly metastatic model and healthy MCF10A as a control [40]. However, it is well known that the biology of TNBC and hormone-positive cancers are unique and that using different subtypes to study metastasis may yield less conclusive results. Another strategy includes using metastatic derivatives of cell lines that were generated after serial selection and in vivo passaging, such as the LM2 cell line derived from MDA-MB-231, which has a high propensity to the lung [41]. While these cell lines have significantly contributed to a better understanding of the mechanisms by which cells metastasize to the lung, they originate from the same MDA-MB-231 cell line and have been artificially selected for use in mouse models. Therefore, they do not necessarily represent a broad set of patient cell lines exhibiting different metastatic behaviors. The use of multiple TNBC cell lines of different metastatic potentials within a study helps to capture this population. However, prior to our study, there was no direct comparison and objective quantification of the metastatic potential of each of these cell lines. We focused on six of the most published TNBC cell lines, injected with the same number of cells in the same conditions and mouse model to determine their metastatic potential relative to each other. Our results demonstrate that 231, 468, and BT549 cell lines are highly tumorigenic and metastatic, BT20 is intermediate tumorigenic with poor metastasis to the lungs but metastatic to the livers, SUM159 is intermediate tumorigenic but poorly metastatic to the lungs and livers, and Hs578T is both poorly tumorigenic and metastatic. We believe these data can help rectify the existing discrepancies in the literature, where different cell lines are assigned metastatic potential without sufficient supporting evidence.

Several previous studies have made inaccurate classifications regarding the metastatic potential of these cell lines. In one study, 468 cells were assigned low metastatic potential, while Hs578T, BT549, SUM159, and 231 cells were assigned high metastatic potential [26]. In another study, 231 and SUM159 were labeled as highly metastatic [40]. We believe these misclassifications are likely due to the lack of a comprehensive dataset that directly compares these cell lines in vivo metastatic potential under consistent experimental conditions. Lastly, the recent Metmap study characterized the metastatic capability of 21 basal-like breast cancer cell lines; however, cells were injected intracardially, quantifying exclusively experimental metastasis and not spontaneous metastasis from the primary tumor site [42]. Experimental metastasis relies more on survival in the bloodstream, extravasation, and metastatic outgrowth, while spontaneous metastasis from the mammary gland additionally requires local invasion and intravasation [43]. Interestingly, we saw some similarities and striking differences between the metastatic potential of human TNBC cell lines based on the model used. 231 cells efficiently metastasize to the lung and liver at high rates in both models and BT20s metastasized to the livers but not the lungs from the intracardiac injection, similar to the spontaneous model. However, 468 cells showed poor metastatic potential from the intracardiac injection with most going to the liver, while in our model, these cells metastasized very efficiently to the lung and liver from the mammary gland. With the intracardiac injection, BT549 cells showed no metastasis to any organ, whereas they exhibited robust metastasis to both the lung and liver from the mammary gland in our study, comparable to 231 cells [42]. Overall, our data provide a clear characterization of the metastatic potential of six human TNBC cell lines in a spontaneous metastasis model from the mammary gland. Further, our study demonstrates that the metastatic potential from the primary tumor may be very different from that in an intracardiac injection metastasis model.

Our data suggest that factors characterizing cell morphology are highly predictive of tumor growth and metastatic potential to the lungs and liver. Interestingly, in the 2D adhesion assay the smaller, rounder, and less irregular cell lines 231, BT549, and 468 had larger tumors and more lung and liver metastases while the larger and more irregular cells Hs578Ts and SUM159s had smaller tumors and fewer metastases. These data align with many existing studies that highlight the importance of cell morphology in predicting metastatic behaviors [20]–[22]. Indeed, single-cell clones derived from the 231 cell line demonstrated persistent morphological heterogeneity, which was correlated with metastatic potential in vivo [20]. This study focused less on individual shape factors but instead incorporated data from 216 features characterizing cell and nuclear morphology. Zaritsky et al. used a generative neural network in combination with supervised machine learning to predict the metastatic potential of patient-derived melanoma xenograft and found that pseudopodal extensions were the hallmark properties of metastatic cells [44]. Using murine and human osteosarcoma cells, another study found that cell shape could be used to distinguish the less metastatic cells from the more metastatic cells, with the more metastatic cell lines also smaller, rounder, and less irregular [45]. Many factors can impact cell morphology, and it is important to be mindful of the substrates used when studying the morphology of cultured cells. We have shown that different ECM proteins’ impact on morphology can be used to predict 3D invasion [22]. In our current study, we used Collagen I as a substrate, as it is the most abundant ECM protein in breast tissues [35, 36] In conjunction with existing studies, our data further support the use of cell morphological measurements to dissect the role of individual genes and pathways in metastasis.

Lastly, our study raises some questions about which in vitro cell motility assay and metrics are best to predict metastasis in vivo. Our data show that no single in vitro motility assay significantly correlates with metastasis in vivo. The metric that is most closely associated with lung metastasis was the 3D persistence of single cells invading in Collagen I gel. It is possible that the substrates and local environment used in our studies are not representative enough of the complex ECM found in tissues. Recent advances using decellularized whole ECM scaffolds [36], cell-derived ECM [46], and co-culture systems [47] have sought to better mimic the tumor microenvironment. However, these complex systems make it difficult to parse out the contributing factors in cell behavior compared to simpler systems. In addition, the same cell line once cultured in different labs or under different conditions can evolve into distinct groups [28]. It is also possible that other components of the tumor microenvironment such as stiffness, and factors secreted by resident local and stromal cells, which are absent in these in vitro assays, may also impact the results. Interestingly, when metrics were combined, cell shape metrics or 2D motility and 3D invasion, we could cluster the cell lines of different metastatic potential by PCA (Additional file 1: Fig S3D-E, Fig S4). These data suggest that using multiple in vitro motility assays can offer a more accurate prediction of TNBC in vivo metastasis.

## Conclusions

Overall, we hope this work will provide a helpful resource for the TNBC research community, showing the first direct comparison of metastatic potential among commonly used TNBC cell lines. We encourage researchers to use multiple TNBC cell lines of different metastatic potential to better represent the heterogeneity of the human TNBC population, as well the use of multiple in vitro cell shape and motility assays to study the role of cell-intrinsic and cell-extrinsic features in driving metastasis in TNBC.

### Supplementary Information

**Additional file 1.** Supplemental Material.

## Data Availability

The datasets used and/or analyzed during the current study are available from the corresponding author on reasonable request.

## Abbreviations

ECM: Extracellular matrix
PCA: Principal component analysis
PLSR: Partial least squares regression
TNBC: Triple-negative breast cancer
