# Supplementary material for: Cell morphology best predicts tumorigenicity and metastasis in vivo across multiple TNBC cell lines of different metastatic potential
Source: Breast Cancer Res. 2024 Mar 11;26:43. doi: 10.1186/s13058-024-01796-8 (PMC10929179; doi:10.1186/s13058-024-01796-8)
Supplement: Supplementary file 1 — Additional file 1. Supplemental Material. [file 13058_2024_1796_MOESM1_ESM.docx]

**Supplemental Figures**

**Supplemental Figure 1. Characterization of TNBC cells**

1. Number of papers with each cell line, obtained from PubMed search in June 2023.
2. Western Blot for all 6 TNBC cells and MCF7 cells, stained for HER2, Progesterone receptor (PR), Estrogen receptor α (ER α) and GAPDH as a loading control.
3. Western Blot for all 6 TNBC cells and MCF7 cells, stained for Ecaderin, Vimentin and GAPDH as a loading control.

**Figure S2. Tumor growth of human TNBC cell xenografts implanted in immunocompromised female NOD-SCID mice***.*  Tumor volume over time for each mouse for (A) 231, (B) 468, (C) BT549, (D) BT20, (E) SUM159, (F) Hs578T. (G) Tumor latency for all 6 cell lines. (H) %area of Ki67 positive cells in tumor sections from mice from all cell lines. Data are shown as mean with SEM, n = 8-10 animals/group.

**Supplemental Figure 3. Correlation between tumorigenicity and metastasis in high and low metastatic potential cell lines.** Correlation between tumor volume and the number of lung metastasis for highly (A) and intermediate and low (B) tumorigenic cell lines, tumor volume and size of lung metastasis for highly (C) and intermediate and low (D) tumorigenic cell lines, size of lung metastasis and tumor volume for highly (E) and intermediate and low (F) tumorigenic cell lines. Correlation between tumor volume and the number of liver metastasis for highly (G) and intermediate and low (H) tumorigenic cell lines, tumor volume and size of liver metastasis for highly (I) and intermediate and low (J) tumorigenic cell lines, size of liver metastasis and tumor volume for highly (K) and intermediate and low (L) tumorigenic cell lines.

**Supplemental Figure 4. Characterization of cell line morphology on Collagen I** Quantification of cell shape parameters for each cell line cultured on Collagen I for 4 hrs 231 (n=679 cells), BT549 (n=799 cells), Hs578T (n=538 cells), and SUM159 (n=892 cells) (A) Perimeter (B) Form Factor , and (C) Compactness. Quantification of cell shape parameters for each cell line cultured on Collagen I for 24hrs 231 (n=178 cells), BT549 (n=184 cells), Hs578T (n=91 cells), 468 (n=205 cells), BT20 (n=135 cells) and SUM159 (n=192 cells) (D) Perimeter (E) Form Factor, and (F) Compactness. Significance was determined using a one-way ANOVA with Tukey’s multiple comparison test comparing each cell line to every other cell line. Significance (p<0.05) is denoted by a letter corresponding to each cell line tested, 231 (a), 468 (b), BT549 (c), BT20 (d), SUM159 (e), Hs578T (f).

**Supplemental Figure 5. Characterization of cell line morphology on tissue culture plastic.** Quantification of cell shape parameters for each cell line cultured on plastic for 4hrs 231 (n=679 cells), BT549 (n=799 cells), Hs578T (n=538 cells), and SUM159 (n=892 cells) (A) Area/Cell, (B) Solidity, (C) Eccentricity, (D) Perimeter, (E) Form Factor, and (F) Compactness. Quantification of cell shape parameters for each cell line cultured on plastic for 24hrs 231 (n=178 cells), BT549 (n=184 cells), Hs578T (n=91 cells), 468 (n=205 cells), BT20 (n=135 cells) and SUM159 (n=192 cells). (G) Area/Cell, (H) Solidity, (I) Eccentricity, (J) Perimeter, (K) Form Factor, and (L) Compactness. Significance was determined using a one-way ANOVA with Tukey’s multiple comparison test comparing each cell line to every other cell line. Significance (p<0.05) is denoted by a letter corresponding to each cell line tested, 231 (a), 468 (b), BT549 (c), BT20 (d), SUM159 (e), Hs578T (f).

**Supplemental Figure 6. Characterization and clustering of cell lines 2D and 3D single cell motility** Principal component analysis of the single cell motility metrics for each cell line (A) PC scores of each cell line projected onto PC1 and PC2 (B) loadings scores of each cell morphology metric projected onto PC1 and PC2.

**Supplemental Figure 7. Distinct differences in cell line-specific morphology on Collagen I and plastic for 4 or 24hrs*.*** (A) pairwise analysis of the strength of the linear relationship (R^2^) between *in vitro* morphology and *in vivo* behavior. Colors represent R^2^ value where blue is high and white is low indicated by gradient (B) Spearman correlation between *in vitro* morphology and *in vivo* behavior. Colors represent the Spearman correlation coefficient where blue is a high positive correlation, white is no correlation and red is a negative correlation indicated by gradient (C) Pearson correlation between *in vitro* morphology and *in vivo* behavior. Colors represent the Pearson correlation coefficient where blue is a high positive correlation, white is no correlation and red is a negative correlation indicated by gradient (D) X-scores plot of the PLS model (E) PLS loadings for adhesion and *in vivo* tumor volume, lung and liver metastases.

**Supplemental Figure 8. Pearson correlation between *in vitro* metrics and *in vivo* behavior.**
